# Diagnosis and Screening for Obesity-Related Conditions Among Children and Teens Receiving Medicaid — Maryland, 2005–2010

**Published:** 2014-04-11

**Authors:** Lee Hurt, Cheryl De Pinto, Johnna Watson, Marti Grant, Julie Gielner

**Affiliations:** 1 Armed Forces Health Surveillance Center; 2 Maryland Department of Health and Mental Hygiene; 3 The Hilltop Institute, University of Maryland, Baltimore County

The prevalence of obesity among children and adolescents in the United States tripled during 1980–2008 and plateaued during 2008–2010 (1,2). This rise in obesity was associated with a rise in chronic conditions previously observed mostly in adults, including hypertension, hypercholesterolemia, and type 2 diabetes (3–6). In 2007, the American Academy of Pediatrics published Expert Committee recommendations for universal screening for overweight and targeted laboratory screening for metabolic disorders among children and adolescents with a body mass index (BMI) at or above the 85th percentile based on age or presence of certain risk factors (7). To assess the prevalence of overweight and obesity among children and teens enrolled in Maryland Medicaid or the Maryland Children’s Health Program (MCHP) and whether or not the children were being screened for obesity-related conditions according to the Expert Committee recommendations, investigators from the Maryland Department of Health and Mental Hygiene computed BMI percentiles for age and sex on a random sample of persons aged 2–19 years enrolled in Maryland Medicaid or MCHP whose height and weight were measured during a well-child visit. Encounter records were used to identify obesity-related conditions and screening laboratory tests received. This study found that 16.5% of participants were overweight (BMI in the 85th–94th percentiles) and 21.4% were obese (BMI at or above the 95th percentile). Obesity was highest among those aged 12–19 years (25.6%) and among Hispanics (28.1%). The diagnosis of obesity-related conditions increased significantly with increasing BMI, with 33.5% of obese participants diagnosed with asthma, 7.9% diagnosed with dyslipidemia, and 7.2% diagnosed with depression. Only 29.9% of overweight and 40.2% of obese participants received a lipid panel test. The results of this investigation were communicated to pediatric, public health, and managed-care leaders. Efforts to communicate the need to increase obesity screening and laboratory testing among this population should continue.

Approximately 383,000 children and teens aged 2–19 years received Medicaid/MCHP Healthy Kids services through the HealthChoice program each year during 2005–2010. From this population, the study sample was drawn by each year selecting at random from those who had managed-care organization encounters during that year. This process yielded approximately 1,600 charts per year that were reviewed by Medicaid Healthy Kids nurses to ensure the visit adhered to the Centers for Medicare and Medicaid Services’ guidelines for early and periodic screening, diagnosis, and treatment (8). During these quality-assurance reviews, the nurses abstracted the child/teen’s height, weight, and date of service, resulting in a final study population of 10,882 children and teens. All height and weight data had been directly measured by the health-care provider during the well-child visit. The data were combined with Medicaid/MCHP enrollment data to get each child/teen’s sex, race/ethnicity, and date of birth needed for computing their exact age at the time of their visit. These data were then used to compute BMI percentile for age and sex using a computer program provided by CDC (9). Each child/teen’s BMI percentile for age and sex was categorized into one of three groups: below the 85th percentile (classified as normal or underweight), in the 85th–94th percentiles (classified as overweight), or at or above the 95th percentile (classified as obese).

The data were linked to Medicaid/MCHP encounter data to gather information about obesity-related comorbidities and health-care provider screening (e.g., laboratory tests and family history). Each child/teen’s visits going back 5 years (or to birth in the case of children aged <5 years) were searched for *International Classification of Diseases, 9th Revision, Clinical Modification* (ICD-9-CM) codes to identify primary and secondary diagnoses of morbidities known to be associated with overweight and obesity.* These codes were also used to identify overweight and obesity-related screening, counseling, and family history–taking performed by the provider. Any screening laboratory tests performed that were related to overweight and obesity were identified through Current Procedural Terminology codes. Statistically significant differences (p<0.05) between groups were determined using Fisher’s exact test and a two-sided Cochran-Armitage test for trend.

Of the 10,882 Healthy Kids study participants, 16.5% were classified as overweight (BMI in the 85th–94th percentiles), and 21.4% were classified as obese (BMI at or above the 95th percentile) (Table 1). The prevalence of obesity increased progressively, from 16.3% in children aged 2–5 years, to 23.1% among children aged 6–11 years, to 25.6% among children and teens aged 12–19 years. No significant difference was observed in obesity prevalence by sex. Hispanic participants had a significantly higher prevalence of obesity (28.1%) compared with their non-Hispanic white (21.0%), non-Hispanic black (20.8%), and non-Hispanic Asian (14.5%) counterparts. No significant change was observed in the prevalence of overweight and obesity during the period 2005–2010.

The rate of screening laboratory tests was lower than expected, based on the recommendations by the Expert Committee for children with elevated BMI (7). The Expert Committee recommends that all children and adolescents with a BMI at or above the 85th percentile for age and sex undergo lipid panel testing, beginning at age 10 years (or if they have other risk factors for comorbid conditions), but only 29.9% of study participants in the overweight category (in the 85th–94th percentiles) were tested, and only 40.2% of participants in the obese category were tested (Table 2). The Expert Committee also recommends that all children and adolescents with a BMI at or above the 95th percentile undergo a fasting glucose test beginning at age 10 years (or if they have other risk factors for comorbid conditions with a BMI in the 85th–94th percentiles); however, only 10.3% of obese study participants underwent this test.

The Expert Committee also recommends that clinicians assess for a family history of overweight and related complications (7). This study found that 1.5% of obese study participants had ICD-9-CM procedure codes for taking a family history of diabetes (Table 2). A similar number were coded for being screened for a family history of lipid disorders. A similar lack of coding occurred for indicating dietary or exercise counseling was provided to obese participants (≤2.0%). The records of few children and teens with a BMI in the 85th–94th percentiles included a diagnosis code of overweight (0.9%). The records of a higher percentage of children and teens with a BMI at or above the 95th percentile included a diagnosis code of obesity (22.3%); however, this is still below the number that met the criteria for obesity based on BMI percentile (7).

What is already known on this topic?

Expert Committee recommendations for the prevention, assessment, and treatment of childhood obesity were released in 2007 that update the 1998 guidelines published by the American Academy of Pediatrics. The recommendations included screening laboratory tests (lipid panel and fasting glucose) for children and adolescents with a body mass index (BMI) at or above the 85th percentile for age and sex, as well as dietary and physical activity assessment and screening for a family history of obesity risk factors.

What is added by this report?

Among Maryland Medicaid or Maryland Children’s Health Program enrollees, the percentage of children and teens aged 2–19 years with a BMI at or above the 95th percentile is higher than in a nationally representative sample of the U.S. population. Despite recommendations for laboratory screening of children and adolescents with a BMI at or above the 85th percentile, the rates of lipid and fasting glucose screening among Maryland Medicaid or Maryland Children’s Health Program enrollees were below what is recommended. Similarly, rates of documented dietary and exercise counseling also were below what is recommended.

What are the implications for public health practice?

Children who are overweight or obese should be appropriately identified and screened for complications, consistent with the Expert Committee recommendations. The increased obesity-related morbidity and low levels of diagnostic coding and laboratory screening identified in this study present a challenge to efforts to reduce and treat childhood obesity. Public health agencies can use this information as an opportunity to assess, understand, and reduce the barriers to implementation of the guidelines.

Diagnoses of medical conditions associated with overweight and obesity were observed to increase significantly across the three BMI groups (Table 2). Asthma, depression, and dyslipidemia were the most common comorbid conditions diagnosed among obese study participants (33.5%, 7.2%, and 7.9%, respectively).

When the data were analyzed to identify emergency department (ED) visits with a primary or secondary diagnosis of an obesity-related complication, the prevalence of these ED visits increased significantly with increasing BMI (Table 2).

## Discussion

This study demonstrates that the prevalence of obesity is higher among Maryland children receiving services through Medicaid/MCHP than in the general population of U.S. children and teens. The prevalence of obesity (21.4%) among the Maryland Healthy Kids study participants was significantly elevated compared with data from the National Health and Nutrition Examination Survey, which includes children and teens with all types of health insurance. In the United States, 16.9% of children and teens aged 2–19 years were categorized as obese during 2009–2010 (2). When stratified by race/ethnicity, the prevalence of obesity among non-Hispanic white Maryland Medicaid/MCHP children and teens was 21.0%, significantly higher than the national rate of 14.0%. Among Hispanic Maryland study participants, the prevalence of obesity was 28.1%, which was significantly higher than the national Hispanic prevalence of 21.2%. No significant difference was observed between the prevalence of obesity among non-Hispanic black children and teens in Maryland (20.8%) and the national prevalence of 24.3%.

This study also indicates these at-risk children and teens are not being adequately coded for overweight and obesity by their Medicaid/MCHP health-care providers and those with a BMI at or above the 85th percentile for age and sex are not receiving recommended screening laboratory tests for obesity-related conditions.

The findings in this study are subject to at least six limitations. First, although the height and weight of each study participant was directly measured by a clinician during a well-child visit, measurement errors or data recording errors might have occurred, resulting in misclassification. Second, because the height and weight were abstracted from a single well-child visit, it is not possible to know when individual participants became overweight or obese or for how long they had been overweight. Third, bias might have resulted because some of the participants were followed for different periods because they were too young to have 5 years of encounter data or because they were enrolled inconsistently in Medicaid in Maryland. Fourth, health-care providers might have screened for overweight and obesity but neglected to record the diagnosis directly in the medical record, and therefore it would not have appeared in the Medicaid/MCHP encounter data used in this study. Fifth, health-care providers might have ordered laboratory tests, but patients might have neglected to follow through with the test. Finally, any screenings or tests performed >5 years before each participant’s chart review were not included in this analysis.

The results of this investigation were presented to the medical directors of all Maryland Medicaid HealthChoice managed-care organizations, the health officers in each Maryland jurisdiction, and pediatricians to make them aware of the need to increase obesity screening and testing for obesity-related complications among this population.

## Figures and Tables

**TABLE 1:** Selected characteristics of children and teens aged 2–19 years participating in the Maryland Healthy Kids study (2005–2010), by body mass index (BMI) percentile

Characteristic	BMI below the 85th percentile			BMI in the 85th–94th percentiles			BMI at or above the 95th percentile			Total no.
		
	No.	%	(95% CI)	No.	%	(95% CI)	No.	%	(95% CI)	
**Total**	**6,769**	**62.2**	**(61.3–63.1)**	**1,790**	**16.5**	**(15.8–17.2)**	**2,323**	**21.4**	**(20.6–22.1)**	**10,882**
**Age group (yrs)**										
2–5	2,746	69.2	(67.8–70.7)	575	14.5	(13.4–15.6)	645	16.3	(15.1–17.5)	**3,966**
6–11	2,165	59.1	(57.5–60.7)	652	17.8	(16.6–19.1)	846	23.1	(21.7–24.5)	**3,663**
12–19	1,858	57.1	(55.4–58.8)	563	17.3	(16.0–18.7)	832	25.6	(24.1–27.1)	**3,253**
**Sex**										
Male	3,429	62.7	(61.4–64.0)	859	15.7	(14.8–16.7)	1,178	21.6	(20.5–22.7)	**5,466**
Female	3,340	61.7	(60.4–63.0)	931	17.2	(16.2–18.2)	1,145	21.1	(20.1–22.3)	**5,416**
**Race/Ethnicity**										
Black, non-Hispanic	3,832	62.8	(61.6–64.0)	1,000	16.4	(15.5–17.3)	1,271	20.8	(19.8–21.9)	**6,103**
White, non-Hispanic	1,865	63.2	(61.4–64.9)	468	15.9	(14.6–17.2)	620	21.0	(19.5–22.5)	**2,953**
Hispanic	595	52.4	(49.4–55.3)	222	19.5	(17.3–22.0)	319	28.1	(25.5–30.8)	**1,136**
Asian, non-Hispanic	201	69.3	(63.7–74.6)	47	16.2	(12.2–21.0)	42	14.5	(10.6–19.1)	**290**

**Abbreviation:** CI = confidence interval.

**TABLE 2:** Number and prevalence of children and teens aged 2–19 years participating in the Maryland Healthy Kids study (2005–2010) who were screened for or received a diagnosis of an obesity-related condition, by body mass index (BMI) percentile and selected characteristics

Characteristic	BMI below the 85th percentile		BMI in the 85th–94th percentiles		BMI at or above the 95th percentile		Total no.	p-value*
		
	No.	Prevalence (%)	No.	Prevalence (%)	No.	Prevalence (%)		
**Total**	**6,769**	**62.2**	**1,790**	**16.5**	**2,323**	**21.4**	**10,882**	
**Diagnosed medical condition**								
Asthma	1,825	27.0	535	29.9	779	33.5	**3,139**	<0.001
Depression	307	4.5	88	4.9	168	7.2	**563**	<0.001
Dyslipidemia	226	3.3	64	3.6	183	7.9	**473**	<0.001
Sleep apnea	98	1.5	33	1.8	77	3.3	**208**	<0.001
Diabetes	44	0.7	21	1.2	58	2.5	**123**	<0.001
Hypertension	43	0.6	13	0.7	61	2.6	**117**	<0.001
Tibia vara	48	0.7	20	1.1	27	1.2	**95**	0.027
Acanthosis nigricans	<6	—†	<6	—†	34	1.5	**40**	<0.001
Overweight	9	0.1	16	0.9	49	2.1	**74**	<0.001
Obesity	33	0.5	70	3.9	519	22.3	**622**	<0.001
Morbid obesity	<6	—†	<6	—†	62	2.7	**70**	<0.001
**ED visit with primary or secondary diagnosis of obesity-related condition**								
Yes	895	13.2	286	16.0	392	16.9	**1,573**	<0.001
**Screening laboratory tests**								
Lipid panel	1,674	24.7	535	29.9	934	40.2	**3,143**	<0.001
Metabolic panel	1,781	26.3	486	27.2	799	34.4	**3,066**	<0.001
Fasting glucose	195	2.9	83	4.6	238	10.3	**516**	<0.001
**Screening for family history and counseling**								
Family history of diabetes	52	0.8	17	1.0	34	1.5	**103**	0.004
Dietary counseling	21	0.3	5	0.3	47	2.0	**73**	<0.001
Exercise counseling	<6	—†	<6	—†	<6	—†	**<6**	0.010
Screening for lipid disorders	45	0.7	16	0.9	33	1.4	**94**	0.001

**Abbreviation:** ED = emergency department.

* Calculated using Cochran-Armitage test for trend.

† Percentages based on fewer than six persons are not shown.

## Footnotes

* Obesity-related conditions included asthma, depression, dyslipidemia, sleep apnea, diabetes, hypertension, tibia vara, acanthosis nigricans, steatohepatitis, dysmetabolic syndrome, and hyperinsulinemia.
